# Photopic and Mesopic Contrast Sensitivity Function in the Presence of Glare and the Effect of Filters in Young Healthy Adults

**DOI:** 10.3389/fpsyg.2021.772661

**Published:** 2021-11-08

**Authors:** Alberto Domínguez-Vicent, Emma Helghe, Marika Wahlberg Ramsay, Abinaya Priya Venkataraman

**Affiliations:** Division of Eye and Vision, Department of Clinical Neuroscience, Karolinska Institute, Solna, Sweden

**Keywords:** functional vision, long pass filter, selective absorption filter, spatial vision, luminance

## Abstract

**Purpose:** The aim of this study was to evaluate the effect of four different filters on contrast sensitivity under photopic and mesopic conditions with and without glare.

**Methods:** A forced choice algorithm in a Bayesian psychophysical procedure was utilized to evaluate the spatial luminance contrast sensitivity. Five different spatial frequencies were evaluated: 1.5, 3, 6, 12, and 18 cycles per degree (cpd). The measurements were performed under 4 settings: photopic and mesopic luminance with glare and no glare. Two long pass filters (LED light reduction and 511nm filter) and two selective absorption filters (ML41 and emerald filter) and a no filter condition were evaluated. The measurements were performed in 9 young subjects with healthy eyes.

**Results:** For the no filter condition, there was no difference between glare and no glare settings for the photopic contrast sensitivity measurements whereas in the mesopic setting, glare reduced the contrast sensitivity significantly at all spatial frequencies. There was no statistically significant difference between contrast sensitivity measurements obtained with different filters under both photopic conditions and the mesopic glare condition. In the mesopic no glare condition, the contrast sensitivity at 6 cpd with 511, ML41 and emerald filters was significantly reduced compared to no filter condition (*p* = 0.045, 0.045, and 0.071, respectively). Similarly, with these filters the area under the contrast sensitivity function in the mesopic no glare condition was also reduced. A significant positive correlation was seen between the filter light transmission and the average AULCSF in the mesopic non-glare condition.

**Conclusion:** The contrast sensitivity measured with the filters was not significantly different than the no filter condition in photopic glare and no glare setting as well as in mesopic glare setting. In mesopic setting with no glare, filters reduced contrast sensitivity.

## Introduction

Ophthalmic filters that block the short wavelength or block selective wavelength are commercially available. Blue light has been linked to cause photochemical damage in the eye (Xie et al., 2014; Lu et al., 2017; Zhao et al., 2018). We are exposed to different natural and artificial light sources in daily basis. Light-emitting diodes (LEDs) are the major light source nowadays in both computer screens and home lighting. These light sources have a significant blue-light component of their spectra. It has been suggested that high levels of blue light from digital devices increase visual fatigue (Coles-Brennan et al., 2019). Hence, the blue light filters have been proposed as a strategy to mitigate eye strain during computer use.

Long-pass filters that filter out the blue light are available in different forms, including spectacle filters (Lawrenson et al., 2017), contact lenses (Cerviño et al., 2008), and intraocular lenses (Zhu et al., 2012; Downie et al., 2018). However, the visual benefits of the filters are quite controversial. Previous studies (Eperjesi et al., 2002; Redondo et al., 2020; Vagge et al., 2021) report findings ranging from no effect to improvement or worsening in visual functions like visual acuity, contrast sensitivity or color vision or in improving eye strain symptoms (Lin et al., 2017; Redondo et al., 2020). In clinical practice, filters are prescribed for subjects with different retinal disorders (Eperjesi et al., 2002) though there is no standard guideline on how to select and prescribe filters.

The discrepancies in previous studies on the effect of filters on visual functions could be due to the differences in filters used, total transmittance of the filter, tests used to measure the visual functions, luminance level and glare level. Photopic contrast sensitivity is shown to reduce systematically, and this reduction has been shown to correlate with the filter transmittance (Leguire and Suh, 1993) in healthy subjects. Most studies use clinically available tests for visual function measurements. Contrast sensitivity tests available for clinical use often contain only a limited level of contrast levels which might not be sensitive enough to detect subtle changes (Pelli and Bex, 2013). Depending on the luminance level, the contrast sensitivity and the effect of filters may vary as there is a marked dissociation between photopic and mesopic contrast sensitivity even in normal observers (Hertenstein et al., 2016). The presence or absence of glare can also have a significant impact on the results on the effect of filters on visual function (Eperjesi and Agelis, 2011).

Contrast sensitivity measurements provide more thorough information on functional vision. The aim of this study is to evaluate the effect of different filters on contrast sensitivity under photopic and mesopic conditions with and without glare. Two long pass filters and two selective absorption filters were used. For the contrast sensitivity measurements, we used an adaptive psychophysical algorithm and evaluated the threshold with a large range of contrast levels.

## Materials and Methods

### Subjects

The measurements were performed in 9 young subjects (aged 18–35 years) with no ocular pathologies. The study design was approved by the regional ethics committee and a written informed consent was obtained from all the subjects after explaining the nature of the study. The study procedures adhered to the tenets of Declaration of Helsinki. The subjects had a best corrected visual acuity of 0.0 logMAR or better. 3 of the subjects were myopes (−0.50 D to −3.00 D), 2 of the subjects was hyperope (+ 1.25 D) and the rest were emmetropes (within ± 0.50 D).

### Materials

Contrast sensitivity measurements were performed with Metropsis research edition (Cambridge Research Systems, United Kingdom). A sinewave grating enveloped in a Gaussian window (Gabor stimulus) of 0.6° standard deviation is used as the visual stimulus. A calibrated 32-inch LCD Display++ Monitor (field of view: 13.25 × 7.50 degrees) with 10-bit resolution was used to present the stimulus and was placed at a distance of 3 m from the subject. The measurements were performed under 4 settings: photopic luminance with no glare (PnG), photopic luminance with glare (PG), mesopic luminance with no glare (MnG), and mesopic luminance with glare (MG). The peak luminance was set at 120 and 3 cd/m^2^ for the photopic and mesopic settings, respectively. Mesopic luminance was achieved using neutral density filter placed on the monitor. The glare source in Metropsis research edition consisted of a pair of intense white LED sources (100 lux) placed above and below the monitor. In order to maintain the same visual angle to the glare source for all the subjects, the center of the monitor was aligned with the subject’s eye level. The glare is directly controlled by the Metropsis system and switches on when the measurements under glare were performed.

**FIGURE 1:**
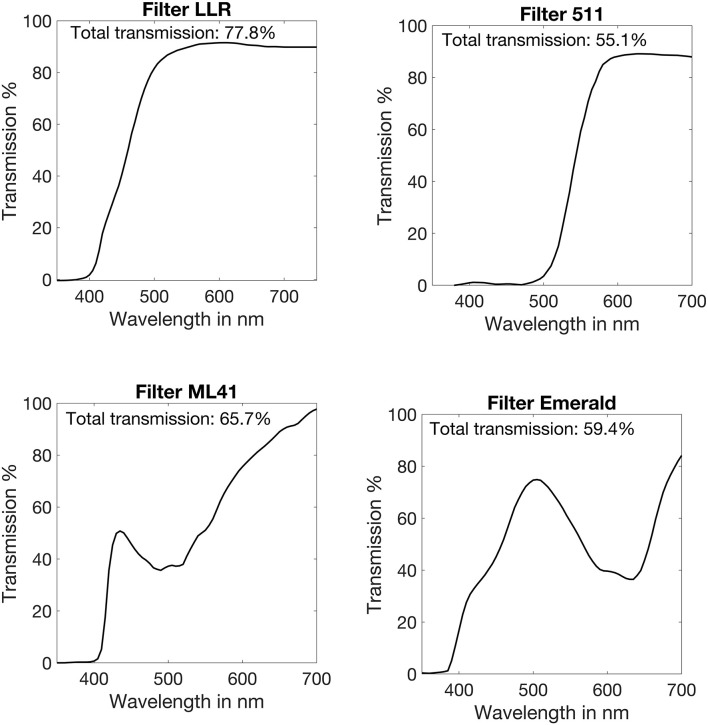
Transmission curves of the filters used in this study. LLR: a long pass LED light reduction filter; 511: 511 nm long-pass filter; ML41: Pink filter; Emerald: Green filter.

In each of the 4 settings, the contrast sensitivity measurements were performed with 4 different filters: (1) a long pass LED light reduction (LLR) filter, designed to reduce the glaring peak in the wavelength spectrum of LED lights, (2) a yellow filter that is a 511nm long-pass filter, (3) a ML41 filter which is a pink filter that has the greatest absorption in the boundary between blue and green light, and (4) an emerald filter which is a green filter that absorbs the short-waved blue light as well as the yellow light. All the filters were from Multilens optical solutions (Multilens AB, Sweden). The total transmission of the four filters is 77.8%, 55.1%, 65.7%, and 59.4%, respectively. The transmission curves of the filters used are given in Figure 1. Control measurements with no filter were also performed in all 4 settings. The average luminance of the stimulus was not corrected for each filter.

### Procedure

The contrast sensitivity measurements utilized a forced choice algorithm in a Bayesian psychophysical procedure. Sixty-four log-equidistant contrast levels ranged between 100% and 0.03% were used. The grating presented was oriented either horizontally or vertically and the subjects were asked to identify the grating orientation and respond with a keypad. Each stimulus was presented for 500 ms accompanied by an auditory cue. No feedback was given about the correctness of the response. The contrast sensitivity measurements were performed for 5 different spatial frequencies: 1.5, 3, 6, 12, and 18 cycles per degree (cpd). The starting contrast for each spatial frequency was set as 65%. All 5 spatial frequencies were interleaved in one measurement set with 40 trials for each spatial frequency. The area under the log Contrast sensitivity function (AULCSF) was calculated for each measurement set.

All the measurements were performed binocularly with natural pupils and the subjects wore their habitual refractive correction. The test conditions and the filter orders were randomized. The filters were worn over the spectacles, if any. All subjects were instructed about the procedure and were given adequate training on the procedure before the measurements. Before the initiation of the first measurement in each condition, the subjects adapted to the respective luminance level for 10 min. Adequate breaks were given between each measurement set and a longer break was given between each condition. The measurements were split into 4 sessions with 1 condition per session. Each session lasted approximately 40 to 45 min.

## Results

**FIGURE 2:**
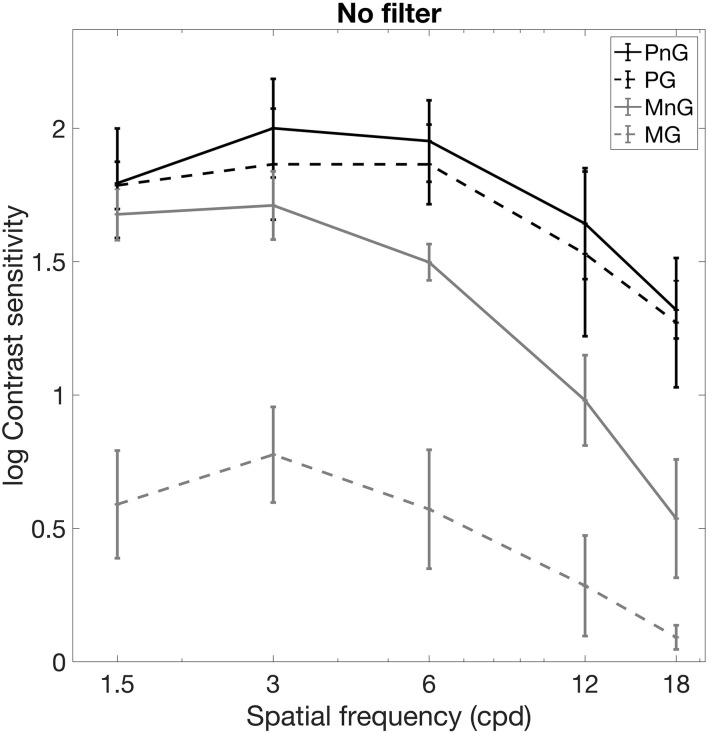
Average photopic and mesopic contrast sensitivity measured without any filter under no glare and glare conditions. PnG: photopic luminance with no glare; PG: Photopic luminance with glare; MnG: Mesopic luminance with no glare; MG: Mesopic luminance with glare. cdp: cycles per degree.

The average photopic and mesopic contrast sensitivity measured without any filter under no glare and glare conditions are shown in Figure 2. A Wilcoxon rank sum test was performed to evaluate the effect of glare at different spatial frequencies. In the photopic setting, the contrast sensitivity values did not show statistically significant difference between the no glare and glare conditions at any of the spatial frequencies (*p* > 0.05 for all 5 spatial frequencies). In the mesopic setting, glare reduced the contrast sensitivity significantly at all spatial frequencies (*p* < 0.0001 for all 5 spatial frequencies).

**FIGURE 3:**
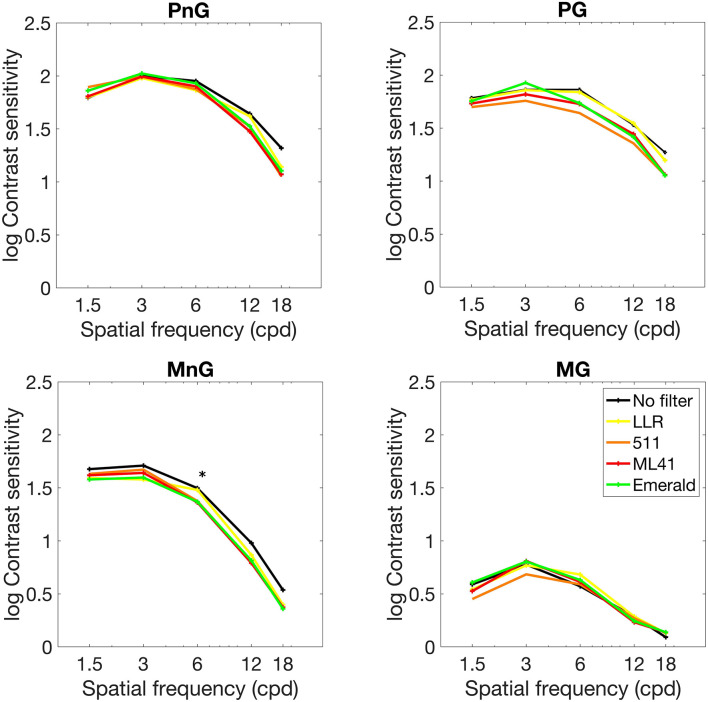
Average photopic and mesopic contrast sensitivity contrast sensitivity measured with different filters under no glare and glare conditions. See Figure 2 legend for the abbreviations. The contrast sensitivity at 6 cpd showed a significant difference with different filter conditions in MnG condition (marked with ^∗^). 511, ML41 and emerald filters reduced the contrast sensitivity values compared to no filter condition.

The average photopic and mesopic contrast sensitivity measured with different filters under no glare and glare conditions are shown in Figure 3. The standard deviation values are not included in the figure due to the number of data points. We evaluated the effect of filters in each condition by performing Friedman non-parametric hypothesis test. At individual spatial frequency level, there was no statistically significant difference between contrast sensitivity measurements obtained with different filters under both photopic conditions and mesopic glare condition. In the mesopic no glare condition, the contrast sensitivity at 6 cpd showed a significant difference with different filter conditions (*X*^2^ = 15.2, *p* = 0.0043). A *post hoc* analysis with Dunn’s multiple comparison test showed that 511, ML41 and emerald filters reduced the contrast sensitivity values compared to no filter condition (*p* = 0.045, 0.045, and 0.071, respectively).

**FIGURE 4:**
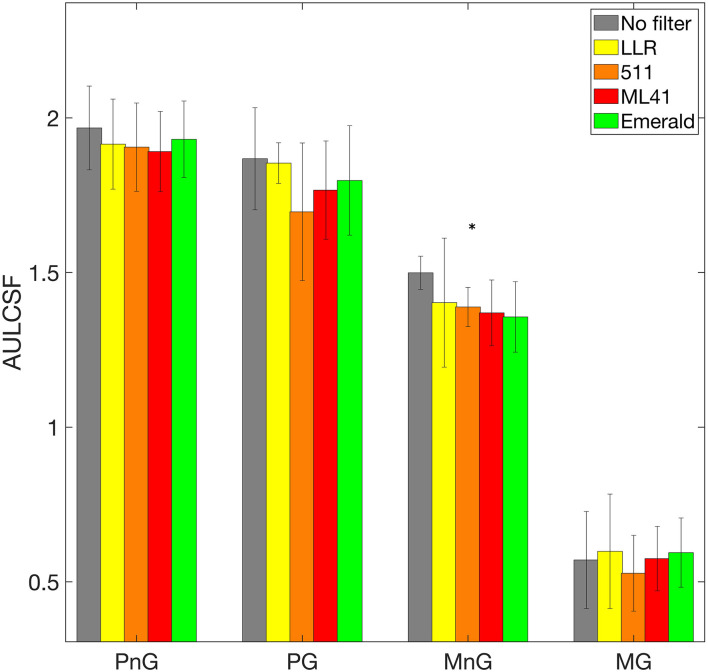
Area under the log Contrast sensitivity function (AULCSF) values for photopic and mesopic settings with no glare and glare condition. See Figure 2 legend for the abbreviations. MnG condition showed a significant difference in the AULCSF values with different filters (marked with ^∗^). 511, ML41 and emerald filters reduced the AULCSF values compared to no filter condition.

Figure 4 shows the AULCSF values for photopic and mesopic settings with no glare and glare conditions. Friedman test showed no statistically significant difference between the AULCSF values obtained with different filters under both photopic conditions and mesopic glare condition. The mesopic no glare condition showed a significant difference in the AULCSF values with different filters (*X*^2^ = 14.58, *p* = 0.0057). A *post hoc* analysis showed that 511, ML41 and emerald filters reduced the AULCSF values compared to no filter condition (*p* = 0.05, 0.024, and 0.009, respectively). A correlation analysis was performed to evaluate the relation between the filter light transmission and the average AULCSF. A significant positive correlation was seen for the mesopic non-glare condition (*r* = 0.915, *p* = 0.029). The other 3 conditions did not show a significant correlation between the filter light transmission and the average AULCSF. Comparing glare and non-glare conditions in the photopic settings, the AULCSF value did not differ significantly for any of the filter except the 511 filter (*p* < 0.0001). In mesopic settings, the AULCSF was significantly reduced with glare compared to no glare condition for all the filters (*p* < 0.0001).

The contrast sensitivity values for individual spatial frequencies and the AULCSF values for each filter were compared between the glare and no glare conditions with Wilcoxon rank sum test. In the photopic setting, all filters showed a small reduction with glare, but only the LLR (at 6 and 12 cpd) and 511 (at 6, 12, and 18 cpd) showed a statistically significant reduction (*p* < 0.05). Similarly, the AULCSF value also reduced with glare for all the filters, with the 511 filter showing a statistically significant reduction (*p* < 0.0001). In the mesopic setting, the contrast sensitivity was significantly reduced with glare for all 4 filters at all spatial frequencies. The AULCSF was significantly reduced with glare compared to no glare condition for all the filters (*p* < 0.0001).

## Discussion

We evaluated contrast sensitivity at 5 spatial frequencies in photopic and mesopic settings with and without glare. The measurements were performed with 4 different filters and no filter condition in a group of young healthy adults. In the photopic conditions with and without glare and the mesopic condition with glare, there was no difference in the contrast sensitivity values between different filters. In mesopic condition with no glare, the contrast sensitivity at 6 cpd showed a significant difference with different filter conditions.

In subjects with low vision, filters have been shown to improve visual functions (Rosenblum et al., 2000; Langagergaard et al., 2003; Colombo et al., 2017) and/or subjective comfort. However, young subjects with healthy eyes do not show similar improvements (Mahjoob et al., 2016; Leung et al., 2017). In some previous reports, the filters have been shown to even reduce visual acuity and contrast sensitivity in younger subjects (Leguire and Suh, 1993; Eperjesi and Agelis, 2011) under both glare and no glare conditions. This reduction is shown to be related to the total transmission of the long-pass or the neutral density filters. In the present study, in addition to 2 long-pass filters (LLR and 511 filters), we also evaluated 2 other filters (ML41 and Emerald filters) that absorb different parts of the light spectrum. Similar to the previous reports, a positive correlation was seen between the filter light transmission and the average AULCSF but only in the mesopic non-glare condition. The four filters evaluated in this study had different transmission, and the stimulus luminance was not modified to match the luminance across filters. As the aim of this study was to evaluate the effect of different filters under photopic and mesopic viewing conditions, we did not adjust the stimulus luminance to keep it constant with different filters.

While comparing the contrast sensitivity with different filters under the same luminance and glare condition, the LLR filter (which absorbs light close to the glaring peak wavelength of LED) was the only filter that showed no difference compared to no filter condition. The other 3 filters (511, ML41 and emerald) showed a significant reduction compared to no filter condition in one of the tests setting (mesopic non-glare) at 6 cpd. Though the total transmittance of the filter plays a role in the change in visual functions, the selective absorption at specific wavelength can determine the visual outcome. The only differences observed were at 6 cpd in the mesopic non-glare condition, and this region is close to the peak of the CSF. The reduced luminance is known to reduce the peak sensitivity and shit the peak toward lower spatial frequencies.

Though the effect of glare in vision increases with age, even younger subjects with no ocular disorders show a reduction in contrast sensitivity in mesopic condition with glare (Puell et al., 2004; van den Berg et al., 2009). In the present study, a reduction in contrast sensitivity with glare was seen in both photopic and mesopic conditions. The reduction in mesopic condition with glare was much larger than the photopic condition. The measurements of contrast sensitivity under mesopic conditions have been suggested to be more valuable than the photopic measurements (Hertenstein et al., 2016). Good contrast sensitivity in mesopic condition can predict good contrast sensitivity in photopic condition, however, the reverse is not always true, even in subjects with no ocular disorders (Koefoed et al., 2015; Hertenstein et al., 2016).

The present study evaluated the effect of different filters (long pass and selective absorption) under photopic and mesopic conditions with and without glare in young subjects with no ocular disorders. Based on the present results, the filter transmission in mesopic non-glare condition seemed to have an impact in the contrast sensitivity values. In elderly subjects and in subjects with different ocular disorders, the effect of long-pass filters on different visual functions has been previously investigated. It would be interesting to evaluate in these groups of subjects the impact of filters that have selective absorption at specific wavelengths.

## Conclusion

The contrast sensitivity measured with long pass and selective absorption filters was not significantly different than the no filter condition in photopic glare and no glare setting as well as mesopic glare setting. In mesopic setting with no glare, filters reduced contrast sensitivity in young healthy adults. The findings can be related to both the total transmittance and the selective absorption of the filter. Glare in mesopic condition significantly reduced the CSF.

## Data Availability Statement

The original contributions presented in the study are included in the article/supplementary material, further inquiries can be directed to the corresponding author.

## Ethics Statement

The studies involving human participants were reviewed and approved by Stockholm Regional Ethics Committee 2021-00176 0311. The patients/participants provided their written informed consent to participate in this study.

## Author Contributions

AD-V, MW, and AV contributed to the conception and design of the work. EH contributed to the data acquisition. AD-V, EH, and AV performed analysis and data interpretation. AD-V and AV drafting of the work. EH and MW revised the draft. All authors contributed to manuscript revision, read, and approved the submitted version.

## Conflict of Interest

The authors declare that the research was conducted in the absence of any commercial or financial relationships that could be construed as a potential conflict of interest.

## Publisher’s Note

All claims expressed in this article are solely those of the authors and do not necessarily represent those of their affiliated organizations, or those of the publisher, the editors and the reviewers. Any product that may be evaluated in this article, or claim that may be made by its manufacturer, is not guaranteed or endorsed by the publisher.
